# Low content Pt nanoparticles anchored on N-doped reduced graphene oxide with high and stable electrocatalytic activity for oxygen reduction reaction

**DOI:** 10.1038/srep43352

**Published:** 2017-02-24

**Authors:** Zeyu Li, Qiuming Gao, Hang Zhang, Weiqian Tian, Yanli Tan, Weiwei Qian, Zhengping Liu

**Affiliations:** 1 Key Laboratory of Bio-inspired Smart Interfacial Science and Technology of Ministry of Education, Beijing Key Laboratory of Bio-inspired Energy Materials and Devices, School of Chemistry and Environment, Beihang University, Beijing 100191, P.R. China; 2 Institute of Polymer Chemistry and Physics of College of Chemistry, BNU Lab of Environmentally Friendly and Functional Polymer Materials, Beijing Normal University, Beijing 100875, P.R. China

## Abstract

A novel kind of Pt/N-rGO hybrid possessing of low content 5.31 wt.% Pt anchored on the surface of nitrogen doped reduced graphene oxide (N-rGO) evenly was prepared. The Pt has uniformed 2.8 nm diameter and exposed (111) crystal planes; meanwhile, the N works as the bridge between Pt and rGO with the Pt-N and N-C chemical bonds in Pt/N-rGO. The Pt/N-rGO material has a very high electrocatalytic activity in oxygen reduction reaction with the mass catalytic activity more than 1.5 times of the commercial Pt/C due to the synergistic catalytic effect of both N-doped carbon matrix and Pt nanoparticles. Moreover, the Pt/N-rGO exhibits an excellent stability with hardly loss (only 0.4%) after accelerated durability tests of 5000 cycles based on the stable Pt-N-C chemical bonds in Pt/N-rGO, which can prevent the detachment, dissolution, migration and aggregation of Pt nanoparticles on the matrix during the long-term cycling.

Platium particles have shown a good electrocatalytic activity in oxygen reduction reaction (ORR) which is the key reaction over the cathode in fuel cells and/or metal-air batteries, *e.g.*, Li-O_2_ battery, due to the incompletely filled *d*-orbital leading to the moderate adsoption^1^,^2^. The matrixes such as carbon-based materials with large surface areas and high electric conductivities are very important for the Pt catalysts to be loaded on ref. 3. Graphene with a two-dimensional (2D) nanosheet structure demonstrates large surface area, high electric conductivity and high mechanical strength, becoming a good choice as the supporting material for the precious metal catalysts^4^,^5^. In order to further enhance the activity and durability of the catalysts, heteroatom-doped graphene could be used as the supporting material 6,7. Nitrogen was chosen as the important dopant element due to the similar size to carbon and one electron more than carbon in the external shell^8^. Beyond the matrix effect, among the different types of doped N, pyridinic and pyrrolic N as well as the carbon atoms next to them have contributed greatly to the ORR catalytic activity^9^,^10^,^11^,^12^,^13^,^14^. As to the Pt particles, both the particle size^15^,^16^,^17^,^18^ and the crystal faces^19^,^20^ may greatly affect the catalytic activity.

However, there is no report on the ORR electrocatalytic catalyst with the aformentioned comprehensive ideal Pt particle size and the crystal faces as well as suitable matrix. Herein, we design the synthesis of Pt/N-rGO nanohybrid catalyst via a facile solvothermal method. The Pt/N-rGO nanomaterial possesses monodispersed Pt nanoparticles with the diammeter of 2.8 nm and exposed (111) facts on the N-doped reducted graphene oxide (N-rGO) nanosheets, which exhibited high ORR electrocatalytic activity and excellent stability after 5000 cycles.

## Results

**Figure 1:**
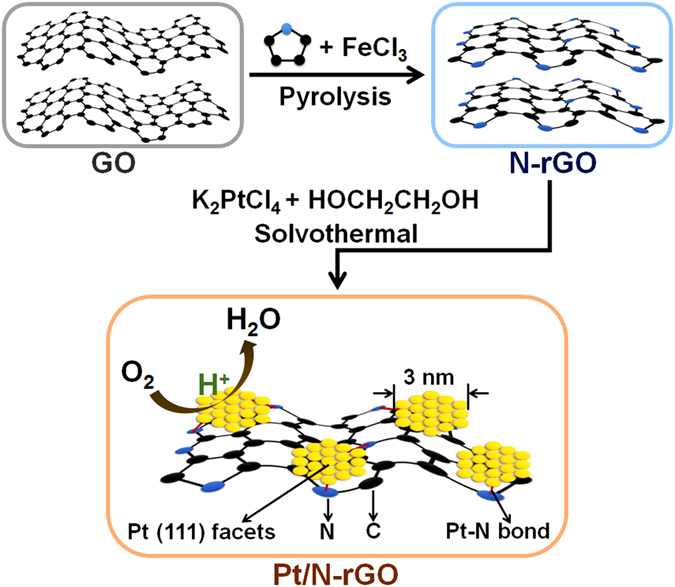
The synthesis and configuration of Pt/N-rGO performed as the catalyst of ORR. The Pt particles with uniformed 2.8 nm diameter and exposed (111) crystal planes anchored on the surface of N-rGO evenly. And the N works as the bridge between Pt and rGO with the Pt-N and N-C chemical bonds in Pt/N-rGO.

**Figure 2:**
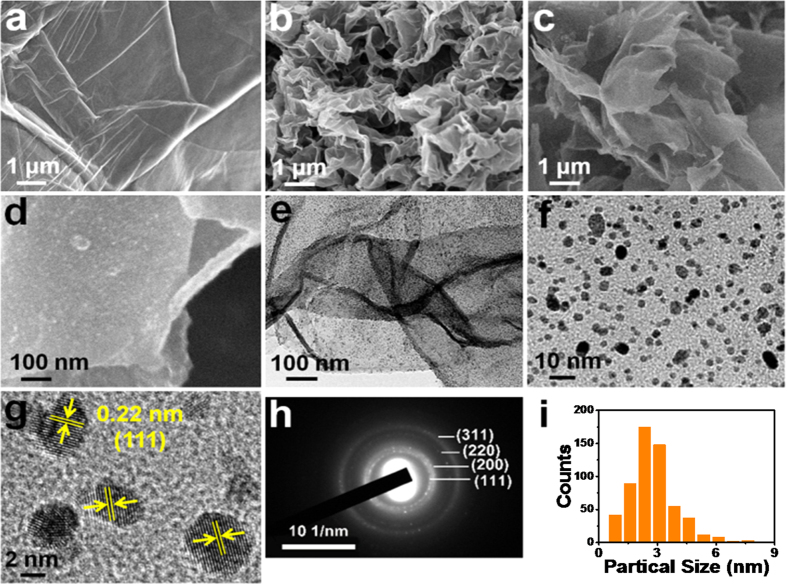
Morphology and structure of GO, N-rGO and Pt/N-rGO. SEM images of GO (**a**), N-rGO (**b**) and Pt/N-rGO (**c**,**d**). TEM (**e**,**f**), HRTEM (**g**) and SAED images (**h**) of Pt/N-rGO. And the histograms of Pt nanoparticle size distribution in (**f**) for Pt/N-rGO (**i**).

The illustration for the synthesis of Pt/N-rGO is shown in Fig. 1. Graphene oxide (GO) was prepared by the modified Hummers method^21^. Pyrrole monomer was then adsorbed on the GO surface and polymerized by dropwise addition of FeCl_3_ solution. After completely polymerized, the sample was subjected to pyrolysis under N_2_ atmosphere to obtain the nitrogen doped reduced graphene oxide (N-rGO)^22^. SEM images of GO and N-rGO are presented in Fig. 2a,b, where the flat and smooth GO changed to the curved but still smooth N-rGO. The Pt nanoparticles were anchored on the surface of the N-rGO under the solvothermal condition with K_2_PtCl_4_ as the Pt source and ethylene glycol as both the reductant and solvent. The Pt loading content was 5.31 wt.% based on the ICP-AES measurement. The morphology and microstructure analyses of Pt/N-rGO were carried out with the SEM and TEM images shown in Fig. 2c–f. The surface of Pt/N-rGO is very flat based on the SEM image observation in Fig. 2c. No large particles could be found from the enlarged SEM (Fig. 2d) and TEM (Fig. 2e) images of Pt/N-rGO. The Pt nanoparticles with the average diameter of about 2.8 nm distributed on the surface of N-rGO nanosheet uniformly were observed on the further enlarged TEM image (Fig. 2f). The related histograms of Pt nanoparticle size distribution may be found for Pt/N-rGO (Fig. 2i). The exposed interplanar distance of 0.22 nm was observed in the HRTEM image of Pt/N-rGO (Fig. 2g) corresponding to the lattice spacing of (111) facets of face-centered cubic (*fcc*) crystalline Pt, which is favor of the ORR electrocatalytic activity^19^,^20^,^23^. The SAED in Fig. 2h presents the polycrystalline structure of Pt/N-rGO with (111), (200), (220) and (311) facets of *fcc*-Pt (JCPDS No. 04-0802). The morphologies and microstructures of the Pt/N-rGO-L and Pt/N-rGO-M samples were also analysized by SEM and TEM techniques for comparison (Supplementary Fig. S1). The Pt particles with average diameter of 2.5 nm (Supplementary Fig. S1a–d) could be observed for Pt/N-rGO-L and some blank places were found on the surface of the matrix due to the less reaction time. The interplanar distances of 0.22 nm were also observed for the Pt nanoparticles in Pt/N-rGO-L and Pt/N-rGO-M (Supplementary Fig. S1c,g), even though the Pt particels of Pt/N-rGO-M became not very uniform with the average diameter increased to 3.5 nm (Supplementary Fig. S1e–h) after the more reaction time.

**Figure 3:**
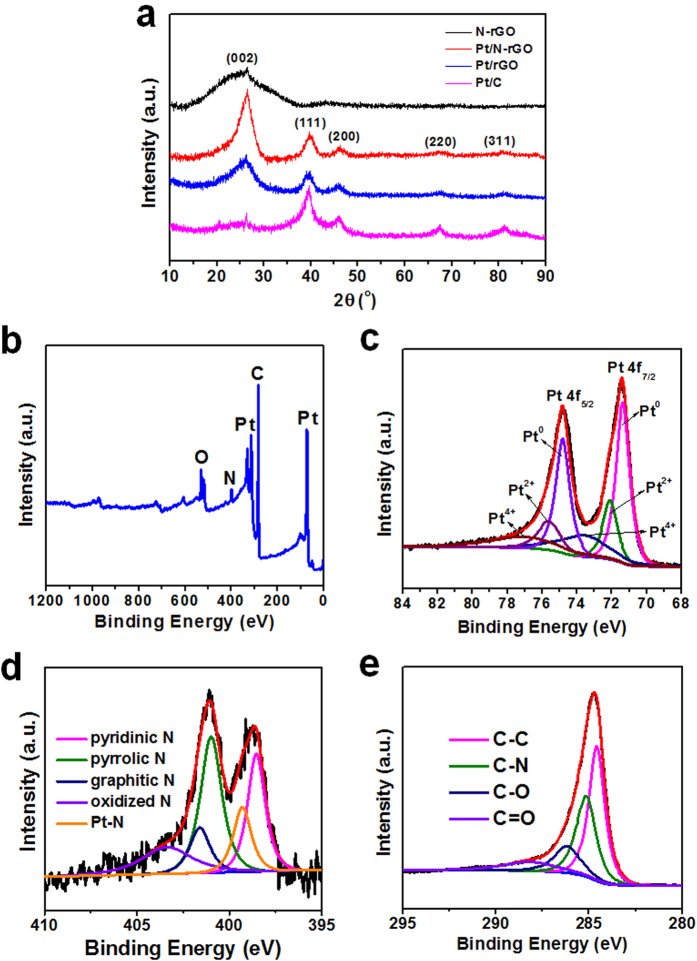
XRD and XPS spectra of N-rGO, Pt/rGO, Pt/N-rGO and the commercial Pt/C. (**a**) XRD patterns of N-rGO, Pt/rGO, Pt/N-rGO and the commercial Pt/C. XPS survey scan spectrum (**b**) as well as the high resolution Pt 4 f (**c**), N 1s (**d**) and C 1s core-level (**e**) spectra of Pt/N-rGO.

XRD patterns are shown in Fig. 3a, where the (111), (200), (220) and (311) facets of *fcc*-Pt could be clearly seen on both Pt/N-rGO and Pt/rGO samples, corresponding to that of the commercial Pt/C. Besides, the (002) facet of graphitic carbon may be obtained for all of the Pt/N-rGO, Pt/rGO and N-rGO samples. XPS survey scan spectrum (Fig. 3b) shows that there are only signals of Pt, O, N and C elements. The Pt 4 f XPS peaks (Fig. 3c) are fitted with three transitions corresponding to the different oxidation states of Pt (Pt^0^, Pt^2+^ as PtO or Pt(OH)_2_ and Pt^4+^ as PtO_2_). Pt^0^, as the main oxidation state in the composite (54.5%), presented a much more content than that of Pt^2+^ (20.9%) and Pt^4+^ (24.6%). The N 1s spectrum (Fig. 3d) suggests the presence of five types of N, *i.e.*, pyridinic N (398.5 eV), pyrrolic N (400.9 eV), graphitic N (401.6 eV), oxidized N (403.4 eV) and Pt-N bond (399.3 eV)^24^. Among them, the main type of plannar pyridinic N (24.7%) and pyrrolic N (34.7%) may possess a low electrical resistance and high electrocatalytic activity of the N-rGO since the nitrogens with planar sp^2^ hybridization would not interrupt the p-p conjugation and avoidan intrinsic barrier impairing a continuous pathway for electron transport. Furthermore, the carbon atoms next to pyridinic and pyrrolic N may be activated where O_2_ molecules could be adsorbed and reduced during the ORR^9^,^10^,^11^,^12^,^13^,^14^. It should be emphasized that the doped N heteroatoms can work as a bridge between the Pt nanoparticles and N-rGO, resulting in the low electrochemical impedance and the excellent stability of the Pt/N-rGO hybrid, which will be discussed later. The formation of C-C, C-N, C-O and C=O bands for Pt/N-rGO are also indicated by the C 1s XPS core-level spectra (Fig. 3e).

**Figure 4:**
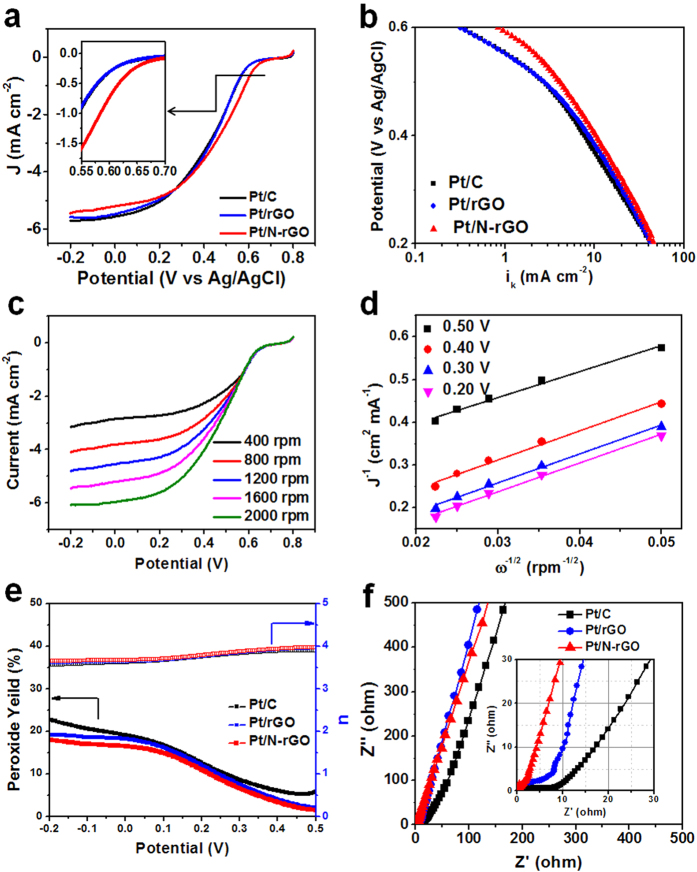
ORR polarization curves of Pt/N-rGO, Pt/rGO and Pt/C. (**a**) The polarization curves of the commercial Pt/C, Pt/rGO and Pt/N-rGO at the rotating speed of 1600 rpm with the insert of the enlarged polarization curves at around the onset potentials for clarity. (**b**) The Tafel plots for Pt/C, Pt/rGO and Pt/N-rGO at the rotating speed of 1600 rpm. (**c**) The rotation rate-dependent ORR polarization curves for Pt/N-rGO. (**d**) The Koutecky–Levich plots from the ORR data at different potentials. (**e**) The peroxide yield with regard to the total oxygen reduction products and the calculated electron transfer number of Pt/C, Pt/rGO and Pt/N-rGO from RRDE in O_2_-saturated 0.1 M HClO_4_. And (**f**) EIS of electrodes of Pt/C, Pt/rGO and Pt/N-rGO.

The activities of the catalysts were measured by RDE operated in O_2_ saturated 0.1 M HClO_4_ with the area of the glassy carbon electrode (GCE) of 0.196 cm^2^ shown in Table 1. Figure 4a shows the polarization curves of Pt/N-rGO, Pt/rGO and the commercial Pt/C with the rotating speed of 1600 rpm. It can be seen that Pt/N-rGO exhibits a much better onset potential of 635.1 mV vs Ag/AgCl than that of Pt/rGO (588.4 mV) and the commercial Pt/C (592.9 mV). The electrocatalytic activity is also approximately estimated by the half-wave potential with the value of 470.1, 440.1 and 430.3 mV vs Ag/AgCl for Pt/N-rGO, Pt/rGO and the commercial Pt/C, respectively, suggesting the best catalytic activitiy of Pt/N-rGO among them. Besides, Pt/N-rGO-L and Pt/N-rGO-M also exhibited better catalytic activities than that of the commercial Pt/C (Supplementary Fig. S2 and Table S1). The onset potentials of Pt/N-rGO-L and Pt/N-rGO-M were 623.7 and 618.4 mV vs Ag/AgCl, which are a little lower than that of Pt/N-rGO. Additionally, the composites of Pt/N-rGO and Pt/rGO also exihibited much better ORR catalytic activity than rGO and N-rGO, indicating the Pt nanoparticlas made most of the contribution to the ORR performance (Supplementary Fig. S3). The kinetic current can be calculated from the ORR polarization curves according to the Koutecky-Levich equation^25^,^26^: 1/*i* = 1/*i*_d_ + 1/*i*_k_, where *i*_d_ is the diffusion-limiting current and *i*_k_ is the kinetic current. The Tafel slope for Pt/N-rGO in Fig. 4b obtained in 0.1 M HClO_4_ was slightly higher than those of Pt/rGO and the commercial Pt/C, with the higher activity at both high and low current density regions depending on the degree of surface coverage of the adsorbed ions. The polarization curves of Pt/N-rGO are shown in Fig. 4c with the Koutecky-Levich plots in Fig. 4d. The value of the number of exchanged electrons was about 4, indicating that Pt/N-rGO follows the four-electron route. The results were also demonstrated by RRDE in Fig. 4e. The amount of H_2_O_2_ producted on the disk electrode was simultaneously detected on the ring electrode. The mole fraction of H_2_O_2_ can be calculated as follows^5^,^27^: 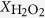 (%) = 2*i*_R_/*N*/(*i*_D_ + *i*_R_/*N*) × 100, where *N* is the collection efficiency (*N* = 0.2), and *i*_R_ and *i*_D_ are the ring and disk current, respectively. The lower content H_2_O_2_ formation over Pt/N-rGO than that of Pt/rGO and the commerical Pt/C corresponded to the number of exchanged electons of about 4. Figure 4f showed the impedance of Pt/N-rGO (0.5 Ω), which was much lower than that of Pt/rGO (5.2 Ω) and the commercial Pt/C (8.5 Ω). The significantly low resistance of Pt/N-rGO demonstrated the tight connection by Pt-N bonds between Pt nanoparticles and N-rGO with a pathway for the electronic transports, which are crucial for the electrochemical activity.

The low content of Pt nanoparticles (5.31 wt.%) anchored on the surface of N-rGO uniformly in Pt/N-rGO resulted in the much better catalytic activity than that of the commercial Pt/C where the content of Pt is 20 wt.%. The high catalytic activity of Pt/N-rGO can be attributed to the apparently synergistic catalytic effect of N-doped carbon matrix^28^,^29^ and the high activated Pt with the very suitable size of about 2.8 nm^15^,^16^,^17^,^18^,^30^,^31^,^32^ which is in favour of the Pt dispersion thus increasing the number of active sites, and overwhelmingly exposed (111) facets with high intrinsic catalytic activity^19^,^20^,^23^. The interaction between C-N and Pt nanoparticles can lead to lower resistance and higher binding energy, decreasing the detrimental strongly adsorbed intermediates, thus leading to the enhancement of the catalytic property^33^,^34^.

**Figure 5:**
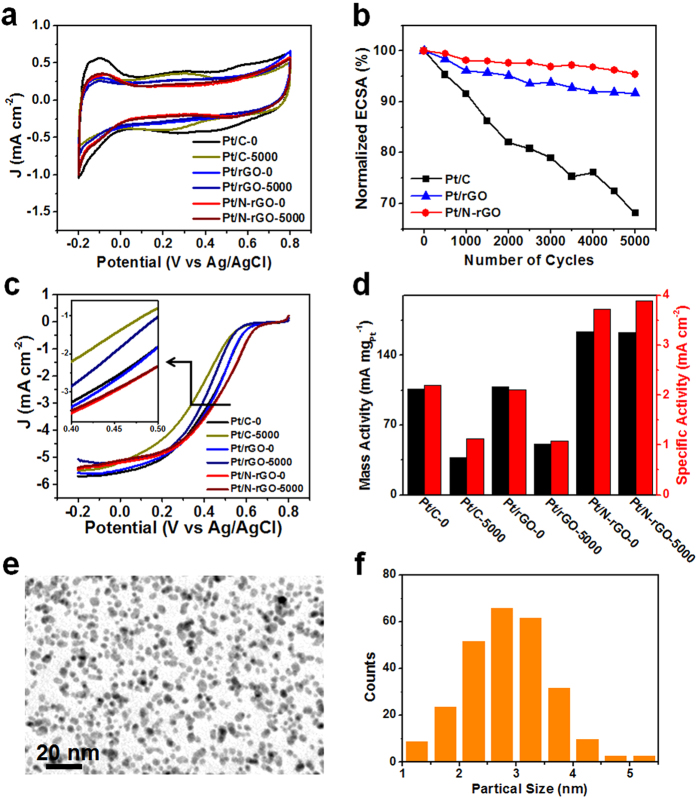
Durability test for Pt/N-rGO, Pt/rGO and Pt/C. (**a**) CV curves of Pt/N-rGO, Pt/rGO and Pt/C before and after ADT of 5000 cycles in N_2_-saturated 0.1 M HClO_4_. (**b**) Comparative ECSA of Pt/N-rGO, Pt/rGO and Pt/C during 5000 electrochemical cycles. (**c**) The polarization curves of the commercial Pt/C, Pt/rGO and Pt/N-rGO before and after the ADT of 5000 cycles in O_2_-saturated 0.1 M HClO_4_with the insert of the enlarged polarization curves at around the half-wave potentials for clarity. (**d**) The mass activities and specific activities of the commercial Pt/C, Pt/rGO and Pt/N-rGO at 0.5 V vs Ag/AgCl. And TEM image (**e**) and the histograms (**f**) of Pt nanoparticle size distribution for Pt/N-rGO after ADT of 5000 cycles.

The stabilities of Pt/N-rGO, Pt/rGO and the commercial Pt/C were investigated by the accelerated durability tests (ADT) at the scan rate of 100 mV s^−1^ for 5000 cycles from 0.2 to 0.6 V vs Ag/AgCl. CV curves were measured in N_2_ saturated 0.1 M HClO_4_ solution at a scan rate of 50 mV s^−1^ before and after ADT of 5000 cycles in Fig. 5a. The electrochemical surface area (ECSA) was calculated by measuring the Coulombic charge for hydrogen desorption in the range of −0.2 to 0.15 V vs Ag/AgCl^35^. The nomalized ECSA evaluated after every 500 cycles are shown in Fig. 5b. The specific values of the ECSA of Pt/N-rGO, Pt/rGO and commercial Pt/C based on the mass of Pt were 65.7, 55.3 and 66.2 m^2^ g^−1^, which are comparable to the typical reported composites of Pt and graphene (Supplementary Table S3). The ECSA of Pt/N-rGO decreased by 4.6%, lower than that of Pt/rGO (8.3%) and much lower than that of the commercial Pt/C (31.9%). The slight drop of ECSA over Pt/N-rGO may be due to the decrease of small amount of Pt^0^, forming some oxide of Pt on the surface recealed by XPS after ADT (Supplementary Fig. S4 and Table S2). Comparing to Fig. 3c, the content of Pt^0^ decreased slightly to 50.9% and Pt^2+^ and Pt^4+^ increased to 22.8% and 26.3%, respectively, thus the oxidation of Pt resulting in the slight decrease of ECSA^36^. The polarization curves of Pt/N-rGO, Pt/rGO and Pt/C before and after ADT of 5000 cycles are also shown in Fig. 5c. The loss of onset potential of Pt/N-rGO is only 1.7 mV, which is much better than that of Pt/rGO (39.4 mV) and the commercial Pt/C (51.1 mV). A slight negative shift of 5.0 mV (only 1.1% loss) was observed on the half-wave potential of Pt/N-rGO, which is better than that of 30.0 mV (6.8% loss) of Pt/rGO and 70.2 mV (16.3% loss) of the commercial Pt/C. The mass and specific activities in Fig. 5d can be obtained by normalizing the kinetic current to the mass of Pt and ECSA, respectively. The mass activity of Pt/N-rGO at 0.5 V vs Ag/AgCl reached 163.4 mA mg_Pt_^−1^,which is much higher than that of Pt/rGO (108.1 mA mg_Pt_^−1^) and the commercial Pt/C (106.0 mA mg_Pt_^−1^). It should be mentioned that the loss of mass activity at 0.5 V vs Ag/AgCl of Pt/N-rGO is only 0.4% after ADT of 5000 cycles, which is much lower than that of Pt/rGO (52.9%) and the commercial Pt/C (65.0%). The specific activity of Pt/N-rGO at 0.5 V vs Ag/AgCl was 3.73 mA cm^−2^, which was retained well to 3.89 mA cm^−2^. For comprison, the specific activity of Pt/rGO (2.10 mA cm^−2^) and Pt/C (2.20 mA cm^−2^) degreased to 1.08 and 1.13 mA cm^−2^, respectively.

TEM images of Pt/N-rGO and Pt/rGO were obtained before and after the ADT. There was no obvious change of Pt/N-rGO after ADT in Fig. 5e. The size distribution of Pt nanoparticles after ADT in Fig. 5f with the mean diameter of 2.9 nm is similar to that before ADT. However, the mean diameter of the Pt nanoparticles on Pt/rGO increased from 3.7 to 5.2 nm after ADT with certain detachment, dissolution, migration and aggregation of Pt nanoparticles (Supplementary Fig. S5). Also, the Pt nanoparticles on the commercial Pt/C aggregated and agglomerated severely into irregular larger nanoparticles after ADT^37^.

The much better stability of Pt/N-rGO than that of the commercial Pt/C and Pt/rGO can be attributed to the doped N working as a bridge between N-rGO matrix and Pt nanoparticles with the Pt-N and C-N chemical bonds. The reported related density function theory calculation also indicates that the binding energy of Pt and the N-doped carbon is much higher than that of the non-doped matrix^38^,^39^,^40^,^41^. Thus, the composite structure of Pt nanoparticles anchored on the N-doped carbon matrix may not only prevent the nanoparticles from detachment, dissolution, migration and aggregation, but also provide a good pathway for the electronic transports, contributing to the improvement in both catalytic activity and stability of the catalyst^37^,^42^. Nitrogen atom with more electrons than carbon atom was doped in the bone of graphite carbon, altering the electron donating character. The plannar N on the edge also provided an extra long pair of electrons enhancing the delocalized π bond and the interaction between Pt nanoparticles and the carbon matrix^36^,^43^.

The ORR performances of Pt/N-rGO were compared with the typical reported composites of Pt and N-doped carbons (Supplementary Table S4). Pt/N-rGO showed the the higher specific activity than most of them due to the synergistic catalytic effect of N-doped carbon matrix and Pt nanoparticles with high activity. The stability of Pt/N-rGO was also better than most of them. The noble metal Pt in Pt/N-rGO takes only 5.31 wt.% of the total weight in Pt/N-rGO, which is one of the lowest among the composites of Pt and N-doped carbon in Tables S3 and S4. In addition, the content of Pt in Pt/N-rGO is also much lower than that of the other usual Pt containing catalysts, *e.g.*, 33 wt.% of Pt/RGO^3^, 24 wt.% of Pt-Rh-Ni/C^19^, 14.3 wt.% of Pt/PANIlong^24^, 37 wt.% of FePtAu^35^, 44.37 wt.% of Pt/CNC(1000)^36^ and 13.01 wt.% of Pt@CN_x_/CNT^37^, and close to that of low content of 5.06 wt.% of TiNiN@Pt^25^. The low loading of Pt in the Pt/N-rGO catalyst may bring about low cost of the catalyst, which is benefit for its commercial utilization.

## Discussions

We have successfully synthesized an unusual ORR hybrid catalyst Pt/N-rGO, in which Pt nanoparticles with uniform size deposited on the surface of nitrogen doped reduced graphene oxide evenly with N working as the bridge between them. The catalyst with low content of Pt demonstrated a much better catalytic performance than the commercial Pt/C due to the synergistic catalytic effect of heteroatom doped carbon matrix and Pt particles with the high activity diameter of uniformed 2.8 nm and exposed (111) crystal planes. The N atoms offered a pathway for the transportation of electrons resulting in the low electrical resistance and prevented the detachment, dissolution, migration and aggregation of Pt nanoparticles on the matrix during the long-term cycling, leading to an excellent stability of the catalyst. Besides, the low loading of Pt in the Pt/N-rGO catalyst may be helpful for getting rid of the constraint of the limited Pt resource in nature, thus promoting its commercial utilization. Such Pt/N-rGO hybrid material possessing of comprehensive ideal Pt particle size and the crystal faces as well as suitable matrix provides a good concept to construct the ORR catalyst with high electroactivity and excellent stability, contributing greatly for the fuel cells and/or metal-air batteries.

## Methods

### Materials preparation

#### GO synthesis

Graphene oxide was prepared as follows. Firstly, 1.0 g of graphite powder was added to 150 mL flask. Then, 24 mL of concentrated sulfuric acid was added and the mixture was stirred at room temperature for 6 h. And then, 0.5 g NaNO_3_ was added to the mixture and stirred for 3 h. After that, 3.0 g of KMnO_4_ was added and the reaction was heated to 45 °C for 30 min. The reaction mixture was further heated to 90 °C in oil bath and held for 30 min after 50 mL of deionized water was added. The obtained mixture was cooled down to room temperature after 100 mL deionized water was dropped in. Finally, 5 mL of H_2_O_2_ (30%) was added in order to complete the oxidation reaction, and the resulting mixture was centrifuged and washed thoroughly with HCl (10%) and water. The obtained precipitate was dispersed in 200 mL of water, which was sonicated for 1 h and freeze dried for 12 h.

#### N-rGO synthesis

30 mg of the prepared GO was dispersed in 35 mL of deionized water and 0.6 mL of pyrrole was dropped into the mixture followed by 5 min of sonication and 10 min vigorous stirring. Then, 25 mL of 0.24 M FeCl_3_ solution was added to the mixture and the stirring was last for 4 h at room temperature to complete the polymerization. The production was separated by filtration and washed with ethanol and water. After dried, the production was subjected to pyrolysis under flowing N_2_ atmosphere at 800 °C for 1 h. Then, the product was immersed in 0.5 M H_2_SO_4_ solution at 80 °C for 8 h to remove the unstable and ORR-nonreactive phases. After washed with deionized water and dried, the sample was pyrolyzed for a second time under flowing N_2_ atmosphere at 800 °C for 3 h.

#### Pt/N-rGO synthesis

Pt nanoparticles were synthesized on N-rGO using a facile solvothermal method. 45 mg of N-rGO was dispersed in 50 mL of ethylene glycol and 10 mL of deionized water. Subsequently, 10 mL of potassium tetrachloroplatinate (II) (K_2_PtCl_4_) aqueous solution (concentration of 1 mg mL^−1^ and aged for at least 24 h) was dropped into the mixture and sonicated for 1 h. Then the mixture was heated to 140 °C with stirring and maintained for 4 h. At last, the product was centrifuged and washed with deionized water and ethanol and dried in a vacuum oven. The product with the reaction time of 4 h was named Pt/N-rGO. The related sample with the reaction time of 2 and 6 h was also obtained and named as Pt/N-rGO-L and Pt/N-rGO-M, respectively, for comparison. The Pt/rGO sample was prepared under the similar experimental conditions as those of Pt/N-rGO with the reaction time of 4 h, where rGO was used as the matrix instead of N-rGO.

### Materials characterization

Scanning electron microscopy (SEM) images were taken on the HITACHI S-4800F with the operating voltage of 5 kV. Transmission electron microscopy (TEM) and high resolution TEM images were obtained on the JEOL JEM-2100F. X-ray diffraction (XRD) patterns were identified on the Panalytical X’Pert Pro X-ray Powder Diffractometer with Cu-Kα radiation. X-ray photoelectron spectroscopy (XPS) curves were examined by the ESCALAB 250Xi of Thermofisher. And inductively coupled plasma atomic emission spectroscopy (ICP-AES) was gotten on the Thermo Scientific.

### Electrochemical measurements

All electrochemical measurements were performed in a three-electrode system on an electrochemical workstation (Pine Instrumentation, Wavedriver 20). The catalysts dispersed on the glassy carbon (GC) rotating disk electrode and rotating ring and disk electrode (RDE and RRDE, Pine Instrumentation) was performed as the work electrode with the geometric area of 0.196 cm and 0.247 cm, respectively. Ag/AgCl (saturated KCl) and Pt wire was used as the reference and counter electrode, respectively. To prepare the work electrode, the catalyst was suspended in the mixture of deionized water, ethanol and 5% of Nafion aqueous solution (v/v/v = 8/1/1) by sonication. The suspension was dropped onto the GC electrode which had been polished with Al_2_O_3_ powder and dried at room temperature. All electrodes were with the mass of loaded Pt of ~25 μg cm^−2^ measured by ICP-AES.

Cyclic voltammetry (CV) curves were conducted with the scan rate of 50 mV s^−1^ from −0.2 to 0.8 V in N_2_-saturated 0.1 M HClO_4_. Linear sweep voltammetry (LSV) measurements with the scan rate of 10 mV s^−1^ were performed with the RDE and RRDE rotation rates varying from 400 to 2000 rpm in O_2_-saturated 0.1 M HClO_4_. The accelerated durability tests were measured at the scan rate of 100 mV s^−1^ for 5000 cycles from 0.2 to 0.6 V vs Ag/AgCl in 0.1 M HClO_4_. The electrochemical surface area of Pt in the catalysts was calculated by the equation: ECSA = *Q*_H_/(*C* × *m*), where *Q*_H_ (μC) is the charge for the hydrogen desorption of the CVs, *C* is the electrical charge (equals to 210 μC cm^−2^) for the monolayer adsorption of hydrogen on Pt nanocrystal surface, and *m* is the mass of Pt loaded on the working electrode. For ORR the mass and specific activity was obtained by normalizing *i*_k_ (obtained from the Koutecky-Levich equation (1/*i* = 1/*i*_d_ + 1/*i*_k_) to Pt and ECSA, respectively. To analyze the number of transferred electrons, the Koutecky-Levich equations were shown as below:


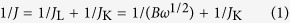



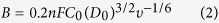



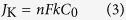


where *J* (mA cm^−2^) is the measured current density, *J*_K_ and *J*_L_ (mA cm^−2^) is the kinetic- and diffusion limiting current density, respectively, *ω* is the angular velocity of the rotating disk (*ω* = 2π*N, N* is the linear rotating speed in rpm), *n* is the overall number of the electrons transferred in oxygen reduction reaction, *F* is the Faraday constant (*F* = 96485 C mol^−1^), *C*_0_ is the bulk concentration of O_2_ (1.26 × 10^−3^ mol L^−1^ in 0.1 M HClO_4_), *D*_0_ is diffusion coefficient of O_2_ (1.7 × 10^−5^ cm^2^ s^−1^ in 0.1 M HClO_4_), *υ* is the kinematic viscosity of the electrolyte (1.009 × 10^−2^ cm^2^ s^−1^ in 0.1 M HClO_4_), and *k* is the electron transfer rate constant. According to equations (1) and (2), the number of transferred electrons (*n*) and *J*_K_ can be obtained from the slope and intercept of the Koutecky-Levich plots, respectively.

## Additional Information

**How to cite this article**: Li, Z. *et al*. Low content Pt nanoparticles anchored on N-doped reduced graphene oxide with high and stable electrocatalytic activity for oxygen reduction reaction. *Sci. Rep.* **7**, 43352; doi: 10.1038/srep43352 (2017).

**Publisher's note:** Springer Nature remains neutral with regard to jurisdictional claims in published maps and institutional affiliations.

## Supplementary Material

Supplementary Information

## Figures and Tables

**Table 1:** The ORR catalytic activities over the Pt/N-rGO, Pt/rGO and Pt/C samples before and after ADT of 5000 cycles measured by RDE operated in the O_2_ saturated 0.1 M HClO_4_.

Factor of the ORR catalytic activity	Sample		
	Pt/N-rGO	Pt/rGO	Pt/C
E_onset_ (mV vs Ag/AgCl)	635.1	588.4	592.9
ΔE_onset_ (mV)	1.7	39.4	51.1
% loss of E_onset_ after ADT	0.3	6.7	8.6
E_half-wave_ (mV vs Ag/AgCl)	470.1	440.1	430.3
ΔE_half-wave_ (mV)	5.0	30.0	70.2
% loss of E_half-wave_ after ADT	1.1	6.8	16.3
Mass activity at 0.5 V vs Ag/AgCl (mA mg_Pt_^−1^)	163.4	108.1	106.0
% loss of mass activity at 0.5 V after ADT	0.4	52.9	65.0
Specific activity at 0.5 V vs Ag/AgCl (mA cm^−2^)	3.73	2.10	2.20
% loss of specific activity at 0.5 V after ADT	−4.3	48.6	48.6
