# Supplementary material for: Low content Pt nanoparticles anchored on N-doped reduced graphene oxide with high and stable electrocatalytic activity for oxygen reduction reaction
Source: Sci Rep. 2017 Feb 24;7:43352. doi: 10.1038/srep43352 (PMC5324168; doi:10.1038/srep43352)
Supplement: Supplementary Information [file srep43352-s1.doc]

*Supplementary information*

**Low content Pt nanoparticles anchored on N-doped reduced graphene oxide with high and stable electrocatalytic activity for oxygen reduction reaction**

Zeyu Li^1,2^, Qiuming Gao^1*^, Hang Zhang^1^, Weiqian Tian^1^, Yanli Tan^1^, Weiwei Qian^1^ & Zhengping Liu^2*^

Key Laboratory of Bio-inspired Smart Interfacial Science and Technology of Ministry of Education, Beijing Key Laboratory of Bio-inspired Energy Materials and Devices, School of Chemistry and Environment, Beihang University, Beijing 100191, P. R. China. Correspondence and requests for materials should be addressed to Q.-M.G. (E-mail: [qmgao@buaa.edu.cn](mailto:qmgao@buaa.edu.cn)).

^2^Institute of Polymer Chemistry and Physics of College of Chemistry, BNU Lab of Environmentally Friendly and Functional Polymer Materials, Beijing Normal University, Beijing 100875, P. R. China. (E-mail: [lzp@bnu.edu.cn](mailto:lzp@bnu.edu.cn)).

**
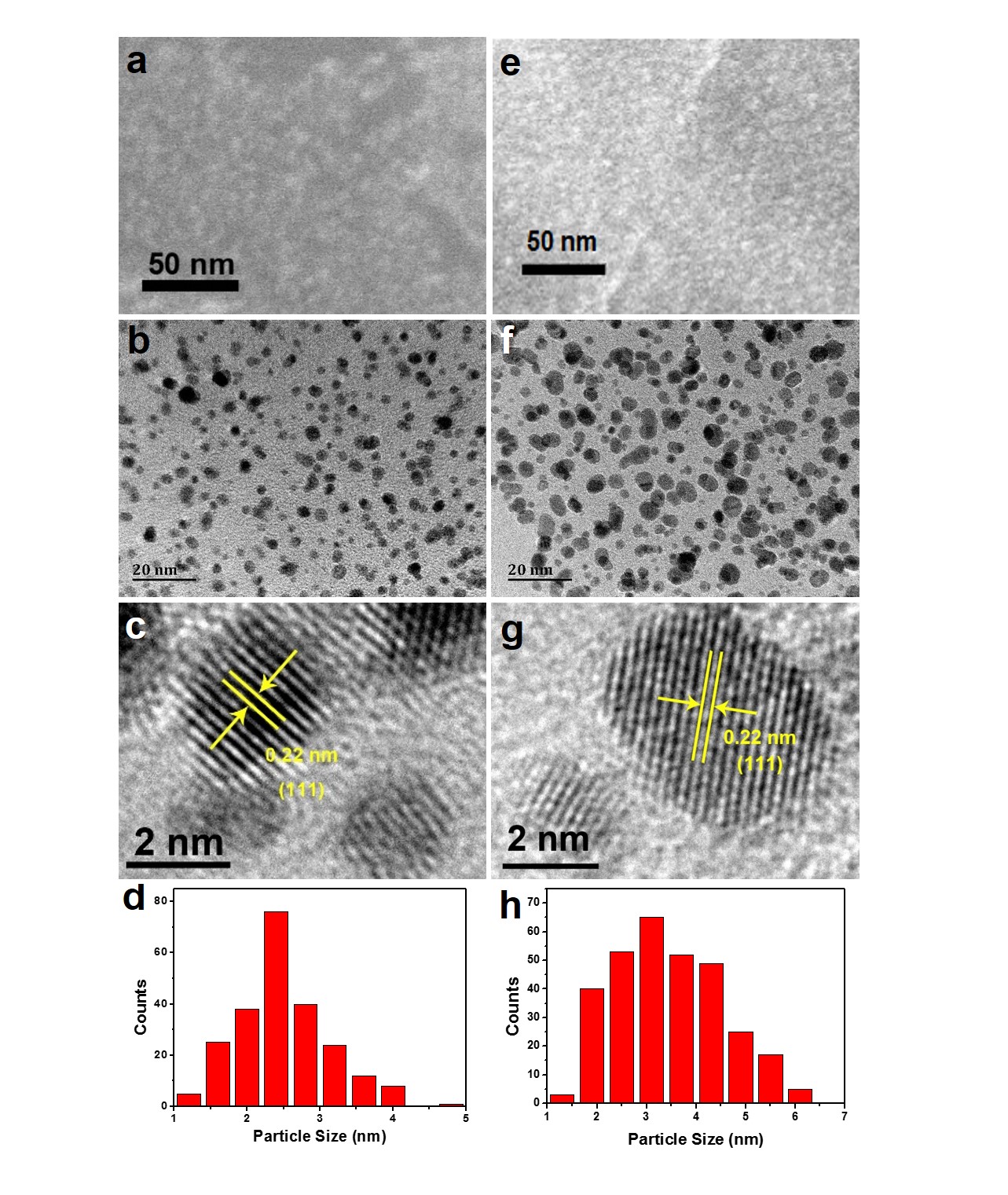
**

**Supplementary Figure S1: Morphologies and structures of Pt/N-rGO-L and Pt/N-rGO-M.** SEM (a and e), TEM (b and f), HRTEM (c and g) images of Pt/N-rGO-L and Pt/N-rGO-M, respectively. And the histograms of Pt particle size distribution for Pt/N-rGO-L (d) and Pt/N-rGO-M (h).

**Supplementary Figure S2: ORR polarization curves.** The polarization curves of the commercial Pt/C as well as the Pt/N-rGO, Pt/N-rGO-L and Pt/N-rGO-M samples at the rotating speed of 1600 rpm. Insert are their enlarged polarization curves.

**Supplementary Figure S3: ORR polarization curves.** The polarization curves of the commercial Pt/C as well as the rGO, N-rGO, Pt/rGO and Pt/N-rGO samples at the scan rate of 10 mV s^-1^ and with the rotating speed of 1600 rpm.

**Supplementary Figure S4: XPS spectrum.** Pt 4f XPS spectrum of Pt/N-rGO after ADT of 5000 cycles.

**
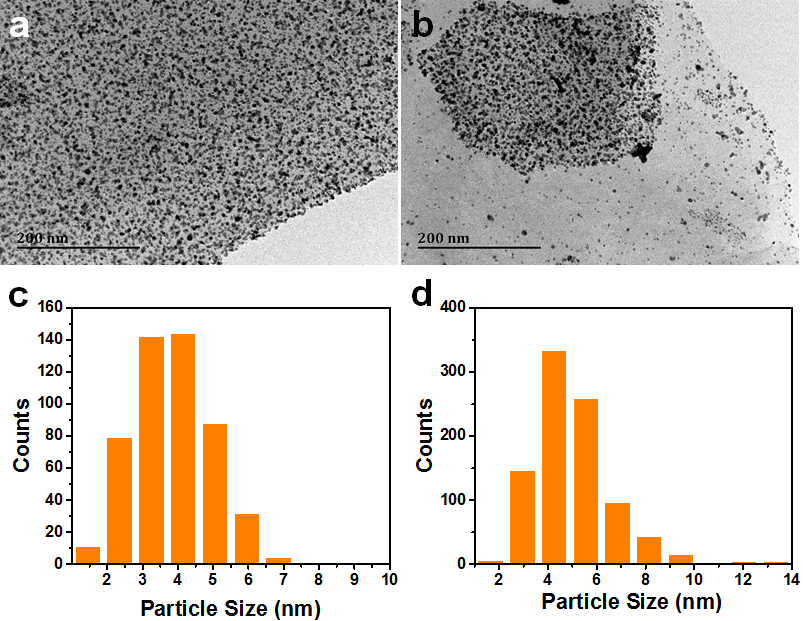
**

**Supplementary Figure S5: Morphologies and structures of Pt/rGO before and after ADT.** TEM image and the histogram of Pt particle size distribution of Pt/rGO before (a and c) and after (b and d) ADT, respectively.

**Supplementary Table S1: Electrochemical catalytic activities of Pt/N-rGO, Pt/N-rGO-L and Pt/N-rGO-M.**

| Sample | Pt/N-rGO-L | Pt/N-rGO | Pt/N-rGO-M |
| --- | --- | --- | --- |
| E_onset_ (mV vs Ag/AgCl) | 623.7 | 635.1 | 618.4 |
| E_half-wave_ (mV vs Ag/AgCl) | 480.1 | 470.1 | 455.2 |

**Supplementary Table S2: Pt sizes and valences recealed by XPS of the initial Pt/N-rGO and after ADT of 5000 cycles.**

| Sample | Size  (nm) |  | Valence | | |
| --- | --- | --- | --- | --- | --- |
|  |  |  | Pt^0^ | Pt^2+^ | Pt^4+^ |
| Initial Pt | 2.8 |  | 54.5% | 20.9% | 24.6% |
| Pt after ADT | 2.9 |  | 50.9% | 22.8% | 26.3% |

**Supplementary Table S3: Comparison of the ECSA of the typical reported Pt/graphene composites with Pt/rGO and Pt/N-rGO.**

| Sample | Pt content (wt.%) | ECSA (m^2^ g^-1^) | Ref. |
| --- | --- | --- | --- |
| Pt/RGO | 33 | 57.0 | 3 |
| Pt@Fe-N/R3DG | 7.1 | 53.4 | S1 |
| Pt/G-PCNF | 20 | 81.0 | S2 |
| Pt/BG3 | 32 | 49.57 | S3 |
| Pt−Ni/GCM | ~15 | 86.7 | S4 |
| Pt/RGO-R | 40 | 34.8 | S5 |
| Pt–Ni/graphene | 20 | 32.0 | S6 |
| Pt/3D-NG | 20 | 52.2 | S7 |
| Pt-Pd nanocubes/RGOs | 37.8 | 50.5 | S8 |
| Pt/PBI-graphene+FCB | 30 | 43.3 | S9 |
| PtG-H | 30 | 101.3 | S10 |
| C-Glu-G/Pt | 13.7 | 74.0 | S11 |
| Au–Pd–Pt NFs/rGO | 26.9 | 44.37 | S12 |
| Pt/S-RGO | 20 | 65.6 | S13 |
| Pt/rGO | 7.80 | 55.3 | This work |
| Pt/N-rGO | 5.31 | 65.7 |

**Supplementary Table S4: Summary of the ORR performances of the composites of Pt and N-doped carbon.**

| Sample | Pt content (wt.%) | Electrolyte | E_onset_  (V) | E_1/2_  (V) | Specific Activity  (mA cm^-2^) | Mass Activity  (mA mg_Pt_^-1^) | Cycle Number | Stability | Ref. |
| --- | --- | --- | --- | --- | --- | --- | --- | --- | --- |
| Pt@CN_x_/CNT | 13.01 | 0.1 M HClO_4_ | - | - | 0.150 | 111.0 | 1500 | 2.3% loss of mass activity | 37 |
| Pt@Fe single bond N/R3DG | 7.1 | 0.1 M KOH | 1.02  vs RHE | 0.88  vs RHE | 2.22 | - | 1000 | 18.1% current decay | S1 |
| Pt NTs/NG | - | 0.1 M HClO_4_ | 1.05  vs RHE | 0.903  vs RHE | 0.771 @0.9 V  vs RHE | 350 @0.9 V  vs RHE | 10000 | 7 mV of ΔE_half-wave_ and 11.1% and 0 loss of mass and specific activity | S14 |
| Pt@NHCS | 20 | 0.1 M HClO_4_ | - | - | 0.41 @0.9 V  vs RHE | 680 @0.9 V  vs RHE | 10800 | ~77% loss of ECSA | S15 |
| CN-Pt/C-10h | 20 | 0.1 M KOH  0.1 M HClO_4_ | -  - | 0.858  vs RHE  - | ~0.43 @0.85 V  vs RHE  0.12 @0.85 V  vs RHE | 214.3 @0.85 V vs RHE  ~80 @0.85 V  vs RHE | 1000 | 10 mV of ΔE_half-wave_ | S16 |
| Pt/CPPA-1400 | 46.9 | 0.1 M HClO_4_ | - | - | 0.324 @0.9 V  vs RHE | 80 @0.9 V  vs RHE | - | - | S17 |
| Pt/NCNC_700_ | 5.1 | 0.5 M H_2_SO_4_ | - | 0.838  vs RHE | 0.94 @0.85 V  vs RHE | 151.7 @0.85 V vs RHE | I-t response | 4% loss after 1 h | S18 |
| Pt/NCB | 20 | 0.1 M HClO_4_ | - | 0.897  vs RHE | 0.107 @0.9 V  vs RHE | 180 @0.9 V  vs RHE | - | - | S19 |
| S3 | 25.10 | 0.1 M HClO_4_ | - | - | 0.834 @0.85 V  vs RHE | 564 @0.85 V  vs RHE |  | - | S20 |
| Pt-SnO_2_/CN_x_/carbon paper | - | 0.5 M H_2_SO_4_ | 0.536  vs RHE | - | 0.015 @0.85 V  vs RHE | 10.836 @0.85 V vs RHE | - | - | S21 |
| Pt–ZrO_2_/NGNs | 14 | 0.5 M H_2_SO_4_ | - | - | - | 102 @0.9 V  vs RHE | 4000 | 51% current decay | S22 |
| Pt/CN_x_/SiO_2_ | 20 | 0.5 M H_2_SO_4_ | - | - | - | 56.9 @ 0.75 V  vs RHE | 500 | ~40 and 60% loss of mass activity at 0.80 and 0.85 V | S23 |
| Pt/N-f-(MWCNT+FLG) | 30 | 0.1 M HClO_4_ | ~1.0  vs RHE | - | 0.76 @0.9 V  vs RHE | 270 @0.9 V  vs RHE | 5000 | 9.6% loss of ECSA | S24 |
| Pt/FeCo–OMPC(L) | 5 | 0.1 M HClO_4_ | - | 0.858  vs RHE | ~0.35 @0.9 V  vs RHE | ~300 @0.9 V  vs RHE | - | - | S25 |
| Pt/HLPC | 20 | 0.1 M HClO_4_ | 0.95  vs RHE | 0.76  vs RHE | 0.0256 @0.80 V vs RHE | 30.9 @0.80 V  vs RHE | - | - | S26 |
| Pt/PCN | 40.3 | 0.5 M H_2_SO_4_ | 0.65  vs Ag/AgCl | 0.52  vs Ag/AgCl | - | 45 @0.55 V  vs Ag/AgCl | - | - | S27 |
| Pt/rGO | 7.80 | 0.1 M HClO_4_ | 0.588  vs Ag/AgCl | 0.440  vs Ag/AgCl | 2.10 @0.5 V  vs Ag/AgCl | 108.1 @0.5 V  vs Ag/AgCl | 5000 | 8.3% loss of ECSA  30.0 mV of ΔE_half-wave_  52.9% loss of mass activity | This work |
| Pt/N-rGO | 5.31 |  | 0.635  vs Ag/AgCl | 0.470  vs Ag/AgCl | 3.73 @0.5 V  vs Ag/AgCl | 163.4 @0.5 V  vs Ag/AgCl |  | 4.6% loss of ECSA  5.0 mV of ΔE_half-wave_  0.4% loss of mass activity |  |

**Supplementary References:**

1. Qin, Y. *et al*. Pt nanoparticle and Fe, N-codoped 3D graphene as synergistic electrocatalyst for oxygen reduction reaction. *J. Power Sources* **335**, 31-37 (2016).
2. Fu, K. *et al.* Facile one-pot synthesis of graphene-porous carbon nanofibers hybrid support for Pt nanoparticles with high activity towards oxygen reduction. *Electrochim. Acta* **215**, 427-434 (2016).
3. Pullamsetty, A. & Sundara, R. Investigation of catalytic activity towards oxygen reduction reaction of Pt dispersed on boron doped graphene in acid medium. *J Colloid Interf. Sci* **479**, 260-270 (2016).
4. Zhou, Y. *et al.* Newly designed graphene cellular monolith functionalized with hollow Pt-M (M = Ni, Co) nanoparticles as the electrocatalyst for oxygen reduction reaction. *ACS Appl. Mater. Interfaces* **8**, 25863-25874 (2016).
5. Sun, K. G., Chung, J. S. & Hur, S. H. Durability improvement of Pt/RGO catalysts for PEMFC by low-temperature self-catalyzed reduction. *Nanoscale Res. Lett.* **10**, 963-969 (2015).
6. Suh, W.-k., Ganesan, P., Son, B., Kim, H. & Shanmugam, S. Graphene supported Pt–Ni nanoparticles for oxygen reduction reaction in acidic electrolyte. *Int. J. Hydrogen Ener.* **41**, 12983-12994 (2016).
7. Zhao, L. *et al.* 3D Hierarchical Pt-Nitrogen-Doped-Graphene-Carbonized Commercially Available Sponge as a Superior Electrocatalyst for Low-Temperature Fuel Cells. *ACS Appl. Mater. Interfaces* **8**, 16026-16034 (2016).
8. Lv, J.-J. *et al.* Facile synthesis of bimetallic alloyed Pt-Pd nanocubes on reduced graphene oxide with enhanced eletrocatalytic properties. *Electrochim. Acta* **143**, 36-43 (2014).
9. Li, Z.-F. *et al.* Hierarchical polybenzimidazole-grafted graphene hybrids as supports for Pt nanoparticle catalysts with excellent PEMFC performance. *Nano Energy* **16**, 281-292 (2015).
10. Vinayan, B. P., Nagar, R. & Ramaprabhu, S. Synthesis and investigation of mechanism of platinum–graphene electrocatalysts by novel co-reduction techniques for proton exchange membrane fuel cell applications. *J. Mater. Chem.* **22**, 25325-25334 (2012).
11. Song, W. *et al.* Carbon-coated, methanol-tolerant platinum/graphene catalysts for oxygen reduction reaction with excellent long-term performance. *J. Mater. Chem. A* **3**, 1049-1057 (2015).
12. Huang, L., Han, Y. & Dong S. Highly-branched mesoporous Au–Pd–Pt trimetallic nanoflowers blooming on reduced graphene oxide as an oxygen reduction electrocatalyst. *Chem. Commun.* **52**, 8659-8662 (2016).
13. Noh, Y. *et al.* Exploring the effects of the size of reduced graphene oxide nanosheets for Pt-catalyzed electrode reactions. *Nanoscale* **7**, 9438-9442 (2015).
14. Zhu, J. *et al.* Strongly coupled Pt nanotubes/N-doped graphene as highly active and durable electrocatalysts for oxygen reduction reaction. *Nano Energy* **13**, 318-326 (2015).
15. Galeano, C. *et al.* Nitrogen-doped hollow carbon spheres as a support for platinum-based electrocatalysts. *ACS Catal.* **4**, 3856-3868 (2014).
16. Lu, L. *et al.* Cyanide radical chemisorbed Pt electrocatalyst for enhanced methanol-tolerant oxygen reduction reactions. *J. Phys. Chem. C* **120**, 11572-11580 (2016).
17. Shrestha, S., Asheghi, S., Timbro, J. & Mustain, W. E. Temperature controlled surface chemistry of nitrogen-doped mesoporous carbon and its influence on Pt ORR activity. *Appl. Catal. A Gen.* **464-465**, 233-242 (2013).
18. Shanmugam, S., Sanetuntikul, J., Momma, T. & Osaka, T. Enhanced oxygen reduction activities of Pt supported on nitrogen-doped carbon nanocapsules. *Electrochim. Acta* **137**, 41-48 (2014).
19. Zhang, S. & Chen, S. Enhanced-electrocatalytic activity of Pt nanoparticles supported on nitrogen-doped carbon for the oxygen reduction reaction. *J. Power Sources* **240**, 60-65 (2013).
20. Balgis, R., Anilkumar, G. M., Sago, S., Ogi, T. & Okuyama, K. Ultrahigh oxygen reduction activity of Pt/nitrogen-doped porous carbon microspheres prepared via spray-drying. *J. Power Sources* **229**, 58-64 (2013).
21. Chen, Y. *et al.* Atomic layer deposition assisted Pt-SnO_2_ hybrid catalysts on nitrogen-doped CNTs with enhanced electrocatalytic activities for low temperature fuel cells. *Int. J. Hydrogen Ener.* **36**, 11085-11092 (2011).
22. Cheng, N. *et al.* High stability and activity of Pt electrocatalyst on atomic layer deposited metal oxide/nitrogen-doped graphene hybrid support. *Int. J. Hydrogen Ener.* **39**, 15967-15974 (2014).
23. Wang, R. *et al.* Highly stable and effective Pt/carbon nitride (CNx) modified SiO_2_ electrocatalyst for oxygen reduction reaction. *Int. J. Hydrogen Ener.* **36**, 5775-5781 (2011).
24. Karthikeyan, N. *et al.* Highly durable platinum based cathode electrocatalysts for PEMFC application using oxygen and nitrogen functional groups attached nanocarbon supports. *Fuel Cells* **15**, 278-287 (2015).
25. Hwang, S. –M. *et al*. Enhancement of oxygen reduction reaction activities by Pt nanoclusters decorated on ordered mesoporous porphyrinic carbons. *J. Mater. Chem. A* **4**, 5869-5876 (2016).
26. Liu, H., Cao, Y., Wang, F. & Huang, Y. Nitrogen-doped hierarchical lamellar porous carbon synthesized from the fish scale as support material for platinum nanoparticle electrocatalyst toward the oxygen reduction reaction. *ACS Appl. Mater. Interfaces* **6**, 819-825 (2014).
27. Su, F. *et al.* Pt Nanoparticles supported on nitrogen-doped porous carbon nanospheres as an electrocatalyst for fuel cells. *Chem. Mater.* **22**, 832-839 (2010).
